# Quantitative trait locus mapping of deep rooting by linkage and association analysis in rice

**DOI:** 10.1093/jxb/erv246

**Published:** 2015-05-28

**Authors:** Qiaojun Lou, Liang Chen, Hanwei Mei, Haibin Wei, Fangjun Feng, Pei Wang, Hui Xia, Tiemei Li, Lijun Luo

**Affiliations:** ^1^Shanghai Agrobiological Gene Center, No. 2901, Beidi Road, Minhang District, Shanghai 201106, PR China; ^2^Fudan University, No. 220, Handan Road, Yangpu District, Shanghai 200433, PR China

**Keywords:** Drought avoidance, genome-wide association study (GWAS), quantitative trait locus (QTL), ratio of deep rooting (RDR), rice, root architecture, selective sweep.

## Abstract

Deep rooting is a very important trait for plants’ drought avoidance, and it is usually represented by the ratio of deep rooting (RDR). Three sets of rice populations were used to determine the genetic base for RDR. A linkage mapping population with 180 recombinant inbred lines and an association mapping population containing 237 rice varieties were used to identify genes linked to RDR. Six quantitative trait loci (QTLs) of RDR were identified as being located on chromosomes 1, 2, 4, 7, and 10. Using 1 019 883 single-nucleotide polymorphisms (SNPs), a genome-wide association study of the RDR was performed. Forty-eight significant SNPs of the RDR were identified and formed a clear peak on the short arm of chromosome 1 in a Manhattan plot. Compared with the shallow-rooting group and the whole collection, the deep-rooting group had selective sweep regions on chromosomes 1 and 2, especially in the major QTL region on chromosome 2. Seven of the nine candidate SNPs identified by association mapping were verified in two RDR extreme groups. The findings from this study will be beneficial to rice drought-resistance research and breeding.

## Highlight

Major quantitative trait loci (QTLs) of rice deep rooting were identified by combined linkage-based and linage disequilibrium-based QTL mapping in this study.

## Introduction

Rice (*Oryza sativa* L.) is a very important crop as it is the staple food for about half the world’s population. However, with the greatest water requirement of all cereal crops, rice often experiences drought due to inadequate rainfall in rain-fed areas (Henry *et al.*, 2012). Furthermore, because of its shallow rooting compared with other cereal crops, rice is particularly susceptible to drought stress, which results in serious yield losses (Kondo et al., 2000, 2003; Uga *et al.*, 2011). Therefore, enhancing drought resistance in rice is a key strategy to stabilize rice production in rain-fed areas. China is experiencing a scarcity of fresh water and frequent drought events, which has led Chinese scientists to launch a new breeding programme to develop water-saving and drought-resistant rice (Luo, 2010).

Drought resistance is related mainly to three aspects: drought avoidance, drought tolerance, and drought recovery (Luo, 2010). Drought avoidance is the first defence against drought stress and plays a main role in enhancing plants’ drought resistance (Blum, 2005). The plants’ roots are the most important organ to absorb and translocate water and nutrients from the soil, so the plants’ ability to avoid drought stress depends mainly on their roots’ performance. Plants with deep rooting are able to access water from deeper soil layers, which enables the plants to avoid drought stress (Yoshida and Hasegawa, 1982; Fukai and Cooper, 1995; Uga *et al.*, 2011). Therefore, modifying the root distribution of rice from shallow rooting to deep rooting is a promising strategy for drought-resistance breeding (Gowda *et al.*, 2011; Uga *et al.*, 2011).

Deep rooting is a complex trait that is determined mainly by a combination of the root growth angle and maximum root length (Abe and Morita, 1994; Araki *et al.*, 2002). At present, the most widely used method to determine deep rooting is the ‘basket’ method. Its evaluation index is the ratio of deep rooting (RDR) (Kato *et al.*, 2006; Uga, 2012). Although many quantitative trait loci (QTLs) responsible for root morphology have been mapped (Courtois *et al.*, 2009), only five major QTLs for deep rooting have been reported (Uga et al., 2011, 2013a, 2015; Kitomi *et al.*, 2015), and only the *DRO1* gene has been cloned, which could improve drought avoidance significantly (Uga *et al.*, 2013b). Most of these reported deep-rooting QTLs were identified from the same deep-rooting variety, Kinandang Patong; however, extensive variations in rice root architecture have been observed (Uga *et al.*, 2009; Table 1) in different varieties, which suggests that there should be more RDR QTLs in the natural material besides the above five.

**Table 1.** Phenotypic description of seven root-related traits in three collections recorded from Hainan, China, in 2013

Trait	Collection 1 (RILs, 180)			Collection 2 (237)			Collection 3 (377)		
	Min	Max	Mean	Min	Max	Mean	Min	Max	Mean
H	61.0	117.0	84.9	65.0	136.0	89.3	49.0	99.7	74.7
T	11.0	74.0	35.0	13.0	76.3	38.8	9.3	60.0	29.5
DR	13.0	231.0	86.7	16.7	250.5	93.9	14.0	170.0	56.8
SR	75.0	606.0	296.4	46.7	671.3	296.0	34.3	470.0	166.0
TR	88.0	750.5	383.1	65.0	904.0	389.9	60.3	586.5	222.8
RDR	10.2%	45.5%	22.7%	3.7%	67.2%	25.0%	4.7%	58.4%	26.8%
TR/T	3.1	19.4	11.2	2.4	23.8	10.6	2.2	20.2	7.9

RIL, recombinant inbred line; Min, minimum; Max, maximum; H, height of shoot (cm); T, number of tillers; DR, number of deep roots; SR, number of shallow roots; TR, total number of roots that penetrate the basket; RDR, ratio of deep rooting (=DR/TR); TR/T, number of roots per tiller.

Most important agronomic traits are quantitative traits and are controlled by many alleles or genes (Ren *et al.*, 2005). Currently, both linkage-based mapping and linkage disequilibrium (LD)-based association mapping are popular methods that enable QTL mapping. Traditional linkage-based QTL mapping has made great progress in identifying important agronomic genes in rice, such as *Gn1a*, which controls grain number (Ashikari *et al.*, 2005), and *GS3*, which controls grain weight and length (Fan *et al.*, 2006). Despite its merits, linkage-based QTL mapping has some limitations. Only the QTLs underlying different phenotypes between the two parents can be found. Furthermore, constructing a suitable population for QTL mapping is labour intensive and time consuming. Conversely, LD-based association mapping uses natural germplasms and there is no need to construct segregating populations. In addition, the invention and wide application of next-generation high-throughput DNA sequencing technologies have greatly facilitated the development of sequencing-based genotyping and genome-wide association studies (GWASs) (Brachi *et al.*, 2011; Huang *et al.*, 2013). GWASs have the potential ability to identify all genes and alleles related to a specific trait but inevitably miss rare alleles (Zondervan and Cardon, 2004; Hirschhorn and Daly, 2005; Wray *et al.*, 2013). Especially for complex quantitative traits, these two gene mining methods cannot always test and verify each other but can be mutually complementary (Mitchell-Olds, 2010; Varshneya *et al.*, 2012). Population genomic approaches involving whole-genome scans for selective sweep regions and single-nucleotide polymorphisms (SNPs) with large frequency imbalances between different groups are also powerful methods to identify useful agronomic genes (Turner *et al.*, 2010; Jiao *et al.*, 2012; Xu *et al.*, 2012).

In the experiment reported here, we carried out a comprehensive study of rice deep rooting in three collections using the ‘basket’ method in the field. We identified some new QTLs and SNPs for RDR through QTL linkage mapping and GWAS analyses. Seven candidate SNPs were verified by Sanger dideoxy sequencing in varieties showing the most extreme RDRs. The findings will enhance our knowledge about the genetic regulation of deep rooting in rice and supply useful information for the breeding of drought-resistant rice.

## Materials and methods

### Plant materials

Three rice collections were used in this experiment. Collection 1 was comprised of 180 F_8_ recombinant inbred lines (RILs), which were developed from Zhenshan97B (lowland *indica* rice variety with shallow rooting) and IRAT109 (upland *japonica* rice variety with deep rooting) (Zou *et al.*, 2005; Yue *et al.*, 2006; Liu *et al.*, 2008). This collection was applied to traditional linkage-based QTL mapping.

Collection 2 consisted of two subsets of rice germplasms: 170 accessions from the mini-core collection of Chinese rice germplasms, provided by Huazhong Agricultural University (Zhang *et al.*, 2011; Chen *et al.*, 2014a), and 67 varieties from the breeding programme of the water-saving and drought-resistance rice (Luo, 2010). Most of the germplasms were Chinese landraces, and GWAS analysis was applied to this collection.

Collection 3 contained 377 landraces from five provinces in China (Xia *et al.*, 2014). The attribute information of the rice landraces, such as subspecies and ecotypes, was provided by the academies that collected them. Twenty accessions from this collection with extreme RDR values were selected for candidate SNP validation.

### Phenotyping

For measurement of deep rooting, all plants were grown in the field at experimental stations in Hainan and Shanghai in China using conventional rice cultivating methods. Collection 1 was evaluated three times: summer 2011 in Shanghai, spring 2012 in Hainan, and spring 2013 in Hainan (for climate and soil conditions, see Supplementary Table S1, available at *JXB* online). Collections 2 and 3 were planted in the spring 2013 in Hainan.

The deep-rooting traits were evaluated using the ‘basket’ method with minor modifications (Uga *et al.*, 2011). The diameters of the top and bottom of the plastic baskets were 17 and 10cm, respectively. The depth of the baskets was 7cm and the basket mesh size was 2mm. All the baskets were filled with soil and sand at a 2:1 ratio (vol:vol) and buried in the field with a distance of 20cm between adjacent baskets (measured from the closest edges of each basket). After germinating in a greenhouse at 28 °C for 12 d, the young seedlings were transplanted into baskets in the fields. Forty days later, the baskets were gently pulled out of the soil. The roots that emerged from the meshes of the baskets were counted. The roots emerging from the bottom and sides were regarded as deep roots (DR) and shallow roots (SR), respectively. The number of tillers (T) and shoot height (H) were also recorded. The total roots (TR=DR+SR), roots per tiller (TR/T) and RDR (=DR/(DR+SR)) were inferred from the SR, DR, and T. Therefore, in total, seven root-related traits were evaluated in this study.

### Genotyping

The genotypes of the RILs were determined using 213 simple sequence repeat markers, as described by Zou *et al.* (2005).

Whole-genome resequencing of collection 2 was conducted using the Solexa Hiseq 2000 system. The raw sequence data have been uploaded to public databases: http://www.ncbi.nlm.nih.gov/bioproject/PRJNA260762 and ftp://ftp-trace.ncbi.nlm.nih.gov/sra/sra-instant/reads/ByRun/sra/SRR/SRR123/SRR1239601. Three pieces of software, BWA (Li and Durbin, 2009), SAMtools, and BCFtools (Li *et al.*, 2009), were used to identify SNPs from clean reads. Finally, 1 019 883 SNP loci were identified. To evaluate the accuracy of the SNPs identified from the original reads, 24 accessions were used for genotypic validation using a high-density whole-genome SNP array, RiceSNP50 (Chen *et al.*, 2014a). Further details of the genomic data processing have been given by Chen *et al.* (2014b).

### Statistical analysis

Analysis of the phenotype data was performed using SPSS version 19 (IBM). Linkage maps were constructed from the genotype data by MAPMAKER/EXP 3.0 software (Lander *et al.*, 1987). QTL analysis was conducted using QTLNetwork (v.2.0) based on the mixed-model based composite interval mapping (MCIM) method (Yang *et al.*, 2007, 2008). An *F*-statistic based on the Henderson method III was used for hypothesis tests. A threshold of *F*>6.4 was used to declare the presence of main-effect QTLs. The threshold was calculated by permutation test (1000 shuffles, 5% significance level) reference to Churchill and Doerge (1994).

The GWAS was conducted using the R statistical package of the Genomic Association and Prediction Integrated Tool (GAPIT) (Lipka *et al.*, 2012), based on the compressed mixed linear model (Zhang *et al.*, 2010). All of the SNPs were included in the association mapping with a 5% minimum allelic frequency (MAF) criterion. The MSU6.0 Nipponbare genome was downloaded from the RGAP database (ftp://ftp.plantbiology.msu.edu/pub/data/Eukaryotic_Projects/o_sativa/annotation_dbs/pseudomolecules/) and used as a reference genome. All analyses of SNP distribution and sequence diversity were completed by in-house scripts in the Linux system.

### Candidate SNP validation

Twenty RDR extreme accessions from collection 3 were chosen for candidate SNP validation. The software Primer Premier v.5.0 (Lalitha, 2000) was used to design primers, and the PCR products were sequenced by Sanger dideoxy sequencing using a 3730xl DNA Analyzer (Shanghai Sangon Biotech Co., China). Clustal X software was used to perform alignment of the sequences (Thompson *et al.*, 1997).

## Results

### Phenotypic analysis

**Fig. 1.**
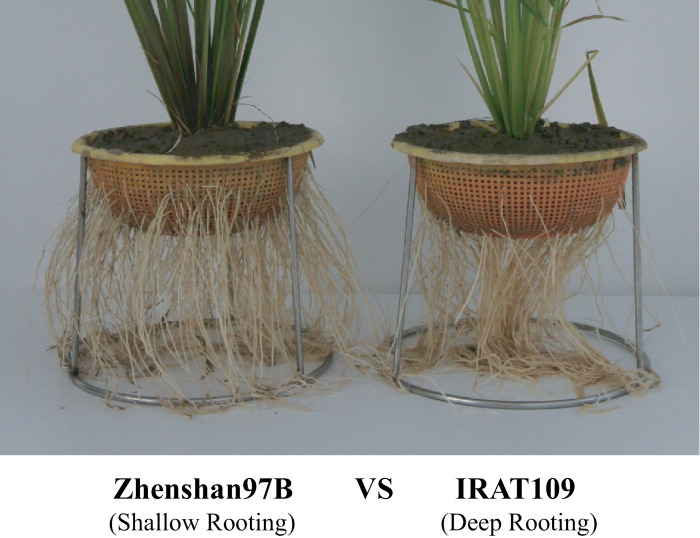
Root architectures of the parents of the RILs.

Figure 1 shows the root architectures of the parents of the RILs: shallow-rooting parent Zhenshan97B (RDR=15.6%) and deep-rooting parent IRAT109 (RDR=47.3%). From the Table 1, it is possible to see the basic rooting traits of collection 1 (RILs), collection 2 and collection 3 from the 2013 Hainan experiment. The RDRs of collection 1 were distributed between the values of the two parental lines and ranged from 10.2 to 45.5%. Collection 2 is a natural population and had wider variation than collection 1 for almost all seven traits with RDRs ranging from 3.7 to 67.2%.

### Linkage-based QTL mapping

A RIL linkage map was constructed with 213 simple sequence repeat markers (Zou *et al.*, 2005). Using this linkage map and the phenotypic data from the three experiments, QTL mapping of RDR was performed (Table 2 and Supplementary Fig. S1, available at *JXB* online).

**Table 2.** Putative RDR QTLs detected by linkage mapping in collection 1

Chr	Interval	F value	A	*P* value	AE1	*P* value	AE2	*P* value	AE3	*P* value
1	RM493–RM157B	16.9	1.70%	1.00E-06	–1.22%	0.03	1.40%	0.01	–0.18%	0.75
2	RM6–RM240	15.8	–3.97%	0.00E+00	0.60%	0.22	–0.67%	0.16	0.07%	0.88
4	RM471–RM119	11.2	2.06%	1.00E-06	0.21%	0.65	0.12%	0.78	–0.33%	0.46
4	RM451–RM317	12.2	–2.65%	0.00E+00	–0.01%	0.93	0.00%	0.96	0.01%	0.90
7	RM478–RM134	6.4	–1.42%	1.50E-05	–0.21%	0.54	–0.06%	0.86	0.27%	0.42
10	RM467–RM596	7.5	1.31%	6.70E-05	0.00%	0.94	0.00%	0.99	0.00%	0.96

Chr, chromosome location of the putative QTLs; *F* value, *F* value of the putative QTLs by *F*-statistic; A, estimated additive effect of the QTLs, a positive A value implies that the P1 parent (Zhenshan 97B) takes a positive value for the additive effect and a negative A value means that the P2 parent (IRAT109) takes a positive value for the additive effect; *P* value, *P* value of the predicted QTL effect; AE1, AE2, and AE3 are the predicted additive effects from the environmental interaction effect in the experiments of 2011sh, 2012hn, and 2013hn, respectively (see Supplementary Fig. S1).

**Fig. 2.**
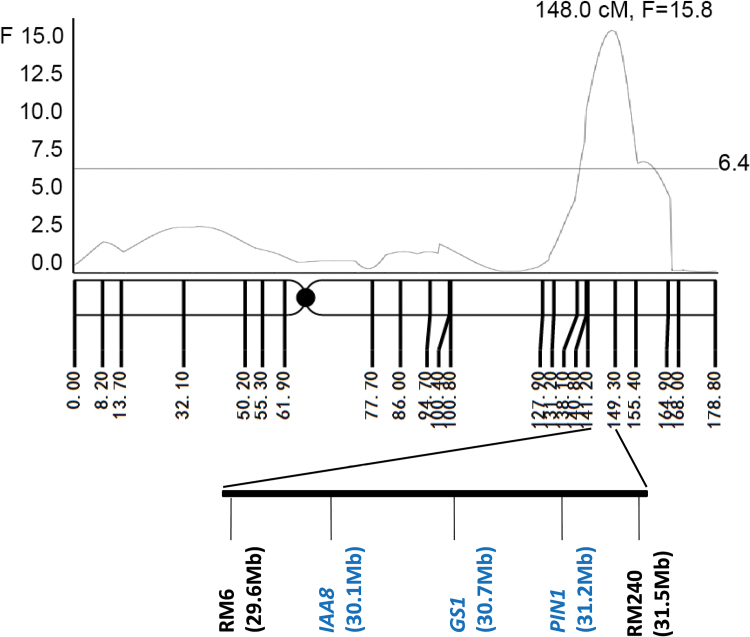
Location of the major QTL on chromosome 2. The peak of the *F* curve indicates the putative position of this QTL. The grey horizontal line (*F*=6.4) indicates the threshold value for this RDR QTL mapping. Vertical lines in the linkage map indicate the genetic position of DNA markers (cM). Flanking markers of the QTL are shown on the bottom; numbers in parentheses beside DNA markers indicate their physical position base on the MSU6.0 Nipponbare genome from the RGAP database. Italic names indicate several known genes located in this region: *IAA8* (LOC_OS02g49160), *GS1* (LOC_Os02g50240), and *PIN1* (LOC_Os02g50960). (This figure is available in colour at *JXB* online.)

A total of six QTLs for RDR were identified from the three experiments, and were located on chromosomes 1, 2, 4, 7, and 10. The deep-rooting parental line IRAT109 provided the positive alleles for deep rooting in three QTLs. A major QTL flanked by RM6 and RM240 on chromosome 2 had the largest additive effect on RDR (Fig. 2). For future work, this QTL was named *qRDR-2* (McCouch *et al.*, 1997).

The environment effect was also calculated, and the variance of environmental effects divided by phenotypic variance [V(E)/V(P)] was 49.70% and the variance of genotype×environment interaction effects divided by phenotypic variance [V(GE)/V(P)] was 2.13%. Environmental factors had an important influence on RDR, but the interaction effect between genotype and environment was not obvious.

Through the QTL mapping analysis of the other traits, the major RDR QTL *qRDR-2* was also found to be related to the SR and TR values (Supplementary Table S2, available at *JXB* online). The allele from ZS97B positively increased the SR and TR values.

### LD-based association mapping

This study used in total 1 019 883 SNPs obtained from genotyping performed on collection 2, and they were distributed at an average of 2.7 SNPs per kb. Most of the SNPs (69.6%) were located in intergenic regions, and only about 13.2% were located in coding DNA sequences.

**Fig. 3.**
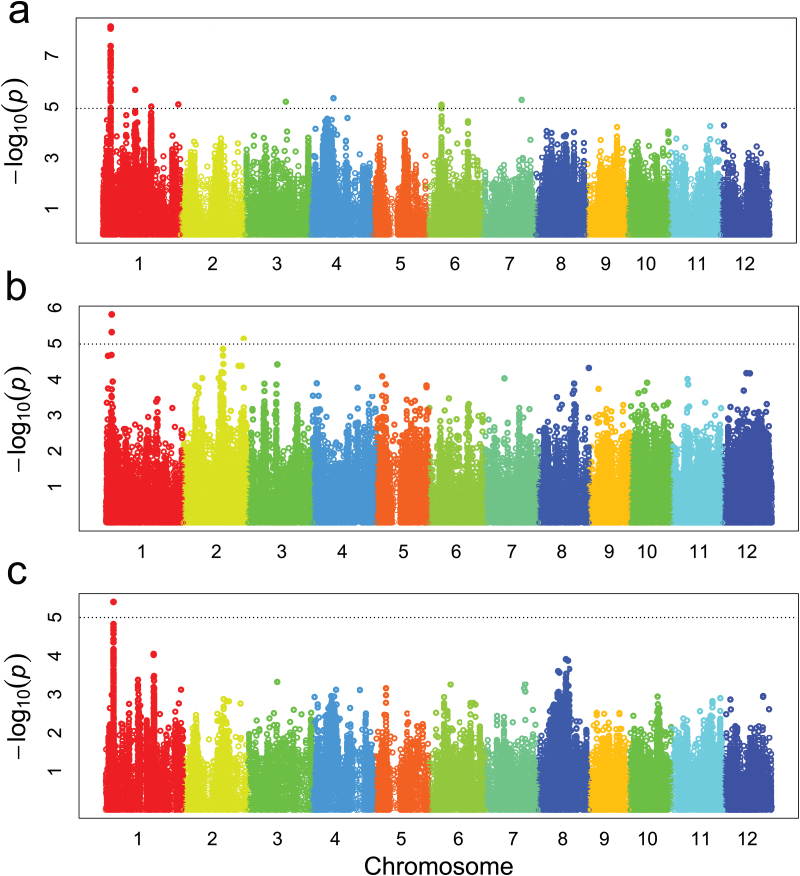
Genome-wide Manhattan plot of the association loci for RDR in collection 2. Association mapping in all 237 rice samples (a), in the *indica* subpopulation (b), and in the *japonica* subpopulation (c). *P* values (–log_10_ transformed) of each test were plotted against the SNP position from whole genome. The horizontal dotted line is the significant level for identification of RDR-associated SNPs.

Using the 1 019 883 SNPs and phenotyping information of 237 varieties, a GWAS analysis of the RDR in collection 2 was performed by GAPIT (MAF>5%). Figure 3 shows the association mapping results in the whole collection (Fig. 3a), in the *indica* subpopulation (Fig. 3b), and in the *japonica* subpopulation (Fig. 3c), respectively. At the end of the short arm of chromosome 1, there was a significant peak in all three groups, and the *P* value of this region calculated from the whole collection was significantly lower than the values calculated from the two subpopulations. In collection 2, 48 associated SNPs (*P*<10^–5^) were identified that clustered into seven regions, which were located on chromosomes 1, 3, 4, 6, and 7. In the *indica* subpopulation, unlike the *japonica* subpopulation or the whole collection, there was a peak (*P*=7.41E–06) on the long arm of chromosome 2, which overlapped with the major QTL *qRDR-2* identified by linakge-based mapping. In total from the *indica* subpopulation, 28 SNPs (*P*<10^–4^) were identified, with most of them being located on chromosomes 1 and 2. Additionally, 24 SNPs (*P*<10^–4^) were found to be related to RDR from the *japonica* subpopulation, and all were located on the short arm of chromosome 1.

### Selective sweep analysis

**Fig. 4.**
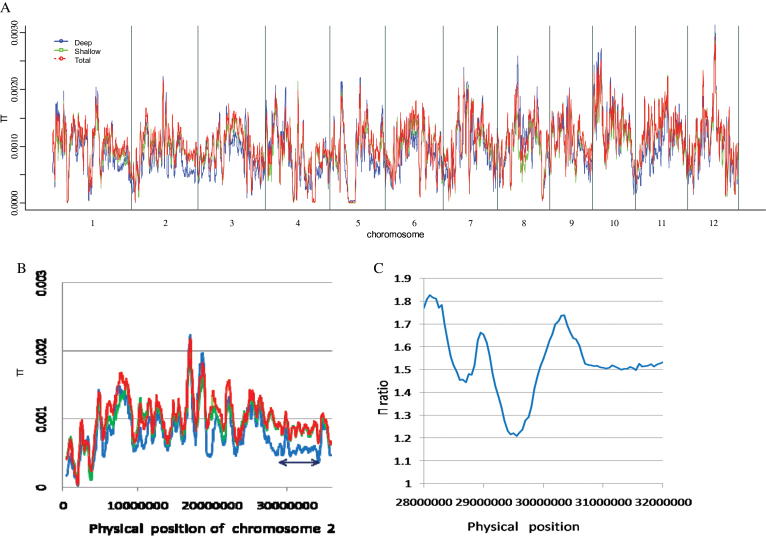
Illustration of the selective sweep signal obtained from collection 2. (a) Nucleotide diversity of the whole rice genome from chromosome 1 to chromosome 12. (b) Nucleotide diversity of chromosome 2. (c) Nucleotide diversity ratio of the major QTL region (from RM6 to RM240 on chromosome 2). π, Nucleotide diversity (number of nucleotide differences per site between two randomly chosen sequences in this population). Sliding 500kb windows were used during the calculation with a 50kb sliding step. The *x*-axis indicates π values. Blue, green, and red lines indicates π values of the deep-rooting group, shallow-rooting group, and complete group, respectively. A clear selective sweep region is indicated by the black arrow. In (c), π ratio=π_shallow_/π_deep._

Selective sweep is a powerful method to find strong selective zones in evolution and to identify important agronomic genes (Lyu *et al.*, 2013). The whole-genome nucleotide diversity of collection 2 and the two extreme RDR groups (shallow-rooting and deep-rooting groups) was calculated using a 500kb sliding window and 50kb sliding step (Fig. 4). Each group consisted of 29 rice varieties with the highest or lowest RDR values from collection 2 (Supplementary Table S3, available at *JXB* online). The average RDR values of the highest and lowest groups were 44.2 and 14.1%, respectively. About 75% of the varieties from the deep-rooting group belonged to the *japonica* subspecies, while 75% of the varieties from the shallow-rooting group belonged to the *indica* subspecies. For the shallow-rooting and the whole collection, the π value (nucleotide diversity=number of nucleotide differences per site between two randomly chosen sequences in this population) distributions were very similar. However, the deep-rooting group had lower nucleotide diversity than the shallow-rooting and complete groups, especially in some regions of chromosomes 1 and 2. Fig. 4b presents the π values for chromosome 2, and there was an obvious selective sweep on its long arm, as indicated by the black arrow. The average π value of the deep-rooting group was 0.000448 in this selective sweep region, while the average π values in the shallow-rooting group and the whole collection were 0.000732 and 0.000685, respectively. Interestingly, the major QTL *qRDR-2* flanked by RM6 and RM240 was located within this selective sweep region. Fig. 4c shows the signal for the π_ratio_ (=π_shallow_/π_deep_) for this QTL region, and all values were greater than 1.2, with the mean of the π_ratio_ for this region being 1.6.

### Candidate SNP validation

**Fig. 5.**
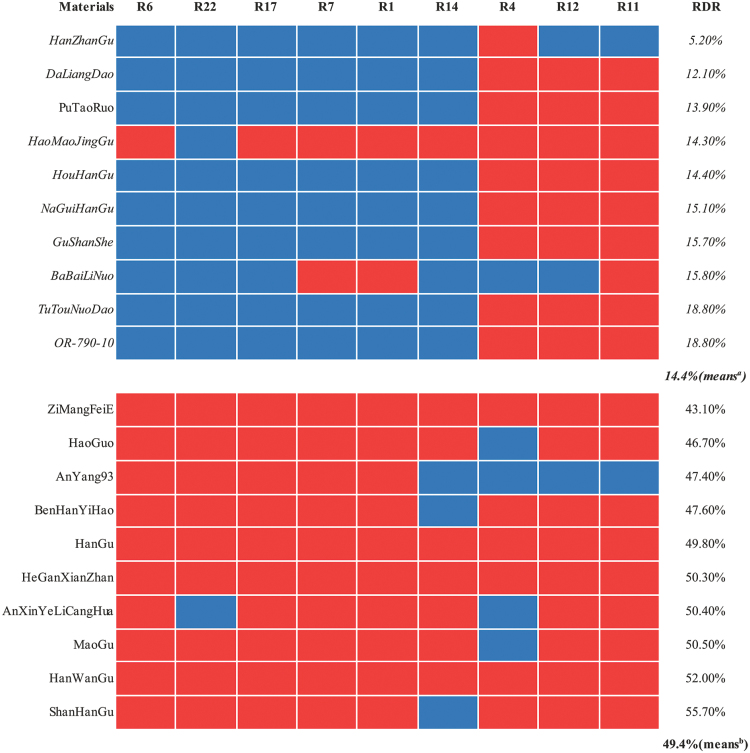
Distribution of nine candidate SNPs in 20 extreme varieties from collection 3. The upper panel shows the results for the extreme shallow-rooting varieties (in italic), and the lower panel shows the extreme deep-rooting varieties. The average values of RDR from the shallow-rooting and deep-rooting groups are given (means^a^ and means^b^, respectively). A red box indicates a major SNP allele type in the deep-rooting group, and a blue box represents another allele type of this SNP.

Collection 3 contained 377 landraces that were used to determine the reliability of the candidate SNPs identified by the GWAS. Twenty landraces with extreme RDR values at the two opposite ends were chosen from this collection for candidate SNP verification. The average values of RDR were 49.4 and 14.4% in the extreme high and low groups, respectively. Nine candidate SNPs were randomly chosen for further verification. Through Sanger sequencing of the PCR products, we obtained sequence information on the candidate SNPs in the 20 RDR extreme landraces (Fig. 5, Supplementary Table S4, available at *JXB* online).

As all nine SNPs could be observed in the varieties used here, this indicated the reliability of the sequence data obtained from the resequencing. Seven of the nine SNPs showed significantly skewed distributions in the two extreme groups, as they did in collection 2. For example, all the deep-rooting varieties possessed the same allele type in the R6 SNP locus, while only one shallow-rooting accession possessed this allele type. According to the allele types of SNPs with skewed distributions, most of the varieties, apart from HaoMaoJingGu, could be classified specifically into two groups: a deep-rooting group and a shallow-rooting group.

## Discussion

Drought resistance is an important but complex trait, and its intensity is determined by the integrative effects of intrinsic and environmental factors, such as root architecture, soil texture, and water and nutrition conditions. As the major organ responsible for water absorption, the roots, especially DRs, play a vital role in plants’ drought resistance (Gowda *et al.*, 2011). However, there have been only a few studies related to deep rooting (Uga et al., 2011, 2013a, b, 2015; Kitomi *et al.*, 2015). To speed up the genetic study of the RDR and to facilitate the breeding of varieties with enhanced drought resistance through marker-assisted selection in rice, a large-scale evaluation of deep rooting in nearly 800 rice accessions was performed and the QTLs/SNPs related to RDR were identified (Table 1, Figs 2 and 3).

In the present study, linkage-based mapping and LD-based association mapping were combined to identify the genetic basis of deep rooting in rice. Six RDR QTLs were identified in three experiments performed under different environmental conditions using the RIL population, and 48 SNPs associated with RDR were detected through LD-based association analysis. However, only a few of the SNPs overlapped with the QTLs identified by linkage-based QTL mapping, and most of them were population specific (Table 2, Fig. 3). The results obtained from linkage-based QTL mapping and LD-based association mapping for complex traits are not identical, but the results can complement each other well (Brachi *et al.*, 2010; Nemri *et al.*, 2010; Famoso *et al.*, 2011). More genes can be identified by the combined use of these two methods.

The first RDR-related QTL (named *DRO1*) was identified by Uga et al. (2011, 2013a), and it could explain 66.6% of the total phenotypic variance. A mutation of *DRO1* was caused by a single 1bp deletion within exon 4 on chromosome 9 in the shallow-rooting parent IR64, and it was only found in several IR64 progeny lines (Uga *et al.*, 2013a). Based on the Sanger sequencing results of this locus in collection 2 and the parents of the RILs, this special mutation does not exist in the materials used in our experiments. However, we found the QTL on chromosome 4 located within a broad interval from RM470 to RM255 that encompassed *DRO2* (Uga *et al.*, 2013b). In addition, the chromosomal regions of the RDR QTL on chromosome 7 (25.95–26.64Mb) and *qSOR1* (24.78–25.59Mb) are very close to each other according to the genome database in Grameme (http://www.gramene.org/). *qSOR1* is the first rice QTL controlling surface rooting, which is the opposite trait to deep rooting (Uga *et al.*, 2012). Recently, Kitomi *et al.* (2015) reported another two QTLs of RDR: *DRO4* and *DRO5*. *DRO4* (28.9–29.9Mb) and *qRDR-2* (29.6–31.5Mb) may be located in the same genomic region. The RIL population used in this work has been used previously to identify QTLs related to panicle number per hill, percentage spikelet fertility, and panicle length, and some of these QTLs coincided with the RDR QTLs located on chromosomes 4 and 7 (Zou *et al.*, 2005; Yue *et al.*, 2006; Liu *et al.*, 2008). The physical interval of the major QTL *qRDR-2* (from RM6 to RM240) was about 1.9Mb, and there are many known genes in this region, such as *IAA8* (LOC_OS49160), *GS1* (LOC_Os02g50240), and *PIN1* (LOC_Os02g50960) (Fig. 2). *IAA8* and *PIN1* both function with auxin (Xu *et al.*, 2005; Jain *et al.*, 2006), and they may take part in the regulation of root distribution. However, further study is needed to clarify their relationship with RDR.

GWAS analysis revealed that some SNPs on chromosome 2 were linked to RDR, but these SNPs were only detected in the *indica* subpopulation (Fig. 3). This might be due to the following reasons. First, according to the phenotypic data (Supplementary Table S5, available at *JXB* online), RDR is significantly related to specific subspecies, in that *japonica* varieties usually have significantly higher RDR values than those of *indica* varieties (Supplementary Table S3), so the genes of RDR within each varietal group may be different. Secondly, *indica* subspecies usually have higher genome diversity than *japonica* subspecies (Huang *et al.*, 2010), which can also be seen in this study where the π value of *indica* (0.000526) was higher than that of *japonica* (0.000369) at the whole-genome level. Most of the associated SNPs on chromosome 2 that were identified only in the *indica* subpopulation belonged to rare allele types in the *japonica* subpopulation, with MAF values of <5%.

The environmental effect on RDR was significant with V(E)/V(P)=49.70%. Therefore, in addition to the genetic effects, RDR may be influenced by many environmental aspects, such as the water regime, nutrition, and degree of soil compaction and composition. Plants that grow in a relatively dry environment might have deeper rooting than those that grow in a well-irrigated environment (Uga *et al.* 2011; Feng *et al.*, 2012). This is a reminder that we need to pay more attention to environmental effects in future studies of RDR.

As well as measuring RDR, we also recorded six other root-related traits (Table 1). By correlation analysis of the seven rooting traits (Supplementary Table S6, available at *JXB* online), it was possible to determine that RDR had a significant negative correlation with the number of tillers. Additionally, fewer tillers and deep-root systems always appeared simultaneously in upland rice (Supplementary Table S7, available at *JXB* online), which often have better drought resistance than lowland rice (Farooq *et al.*, 2009). Therefore, it would be noteworthy to break the linkage drag that might exist between RDR and tiller number when we introduce the deep-rooting genes from the upland variety into the lowland shallow-rooting variety.

In conclusion, we used linkage analysis and association mapping to discover more QTLs for deep rooting. The chromosomal region from RM6 to RM240 included the major QTL (*qRDR-2*) and some associated SNPs, and this region had also undergone strong selection, so this region must be very important for rice deep rooting. Seven of the nine SNPs from the GWAS analysis were verified to be linked with RDR. The authors are currently in the process of map-based cloning of *qRDR-2.* Cloning of *qRDR-2* will provide more insights into understanding the molecular mechanism underlying deep rooting in rice and will facilitate rice variety development with enhanced drought resistance.

## Supplementary data

Supplementary data are available at *JXB* online.

Supplementary Table S1. Basic soil and climate properties at three experimental sites.

Supplementary Table S2. Putative QTLs for SR (shallow root number) and TR (total root number) in collection 1 obtained from linkage mapping using the means of three repeats.

Supplementary Table S3. Two groups of extreme deep and shallow-rooting rice varieties from collection 2.

Supplementary Table S4. Ratios of the two alleles of the candidate SNPs in deep-rooting and shallow-rooting varieties from collection 3.

Supplementary Table S5. Comparison of root traits between different subspecies in collection 3.

Supplementary Table S6. Correlation coefficients among seven root-related traits in all three collections.

Supplementary Table S7. Comparison of root traits between upland and lowland rice in collection 3.

Supplementary Fig. S1. Distribution of RDR (ratio of deep rooting) in RILs. Phenotyping experiments were conducted three times at different locations.: (a) 2011sh in Shanghai, China, in 2011; (b) 2012hn in Hainan, China, in 2012; and (c) 2013hn in Hainan, China, in 2013.

Supplementary Data

## Abbreviations:

GWAS: genome-wide association study
LD: linage disequilibrium
MAF: minor allele frequency
QTL: quantitative trait locus
RDR: ratio of deep rooting
RIL: recombinant inbred line
SNP: single-nucleotide polymorphism
π: nucleotide diversity.
