# Supplementary material for: Quantitative trait locus mapping of deep rooting by linkage and association analysis in rice
Source: J Exp Bot. 2015 May 28;66(15):4749–57. doi: 10.1093/jxb/erv246 (PMC4507776; doi:10.1093/jxb/erv246)
Supplement: Supplementary Data [file supp_66_15_4749__index.html]

Quantitative trait locus mapping of deep rooting by linkage and association analysis in rice — Quantitative trait locus mapping of deep rooting by linkage and association analysis in rice — Supplementary Data 

# Quantitative trait locus mapping of deep rooting by linkage and association analysis in rice

## Supplementary Data

Data files

- Supplementary Data - Supplementary Data
