# Supplementary material for: Quantitative trait locus mapping of deep rooting by linkage and association analysis in rice
Source: J Exp Bot. 2015 May 28;66(15):4749–57. doi: 10.1093/jxb/erv246 (PMC4507776; doi:10.1093/jxb/erv246)

Supplementary Table S1 Basic soil and climate properties at three experimental sites.

Property	Experiments		
Location	Shanghai	Hainan	Hainan
Longitude (˚E)	121.10	110.02	110.02
Latitude (˚N)	31.15	18.48	18.48
Year	2011	2012	2013
Month	Sept-Oct	Feb-Mar	Mar-Apr
Soil type	Clay loam	Sandy soil	Clay loam
Soil pH	6.8	6.2	6.7
Fertilizer N (Kg/ha)	169.7	264.2	208.1
Sunshine duration (h/month)	149.2	104.5	153.4
Average temperature (℃)	21.7	21.8	24.6
Precipitation (mm/month)	36.4	85.5	105.8

The climate data was supplied by China Meteorological Data Center (http://data.cma.gov.cn/data/index/6d1b5efbdcbf9a58.html). The detailed climate data of the experimental sites were referred to the records of the nearest weather station.

Supplementary Table S2 Putative QTLs for SR (shallow root number) and TR (total root number) in collection 1 obtained from linkage mapping using the means of three repeats.

	Chr	Interval	F-value	A	P-Value
**SR** (critical F value=13.8)	1	RM476B-RM315	14.4	-26.0	1.22E-04
	2	RM6-RM240	28.8	43.9	0.00E+00
**TR** (critical F value=13.4)	2	RM6-RM240	16.4	37.4	1.90E-05

Chr: chromosome location of the putative QTLs; F-value: F value of the putative QTLs; A: estimated additive effect of the QTLs, a positive **A** value implies that the P1 parent (Zhenshan97B) takes a positive value for the additive effect and a negative **A** value means that the P2 parent (IRAT109) takes a positive value for the additive effect; P-value: P value of the predicted QTL effect;

Supplementary Table S3 Two groups of extreme deep and shallow rooting rice varieties from collection 2.

Deep rooting group				Shallow rooting group			
**No.**	**Name**	**Subspecies**	**RDR**	**No.**	**Name**	**Subspecies**	**RDR**
S11E0201	DULAR	aus	36.6%	S11E0174	BICO BRANCO	indica	8.8%
S11E0054	LAMBAYEQUE 1	aus	43.7%	S11E0348	MINBEIWANXIAN	indica	10.5%
S11E0157	88B	indica	35.8%	S11E0204	YANGDAO 2	indica	12.2%
S11E0080	WH139	indica	37.3%	S11E0022	XIANGAI B	indica	12.3%
S11E0172	RIKUTO NORIN 21	indica	38.3%	S11E0235	ZHENFU	indica	12.9%
S11E0135	IR 30358-084-1-1	indica	38.9%	S11E0184	GUI 630	indica	13.1%
S11E0026	XIAOHONGGU	indica	43.3%	S11E0207	JINXIBAI	indica	13.2%
S11E0321	HAOBAYONG 1	japonica	35.8%	S11E0318	BAIKEZAOHE	indica	13.4%
S11E0031	SILEWAH	japonica	37.9%	S11E0319	NANTEHAO	indica	13.7%
S11E0253	TAIDONGLUDAO	japonica	39.3%	S11E0328	ZHONG 413	indica	13.9%
S11E0015	ZAOHANDAO	japonica	39.7%	S11E0165	SHANHUANGZHAN 2	indica	14.9%
S11E0043	IAC 1246	japonica	39.8%	S11E0168	SML 81B	indica	15.1%
S11E0005	HAOHAI	japonica	40.1%	S11E0127	DT001	indica	15.3%
S11E0122	GUIHUAHUANG	japonica	40.7%	S11E0215	MEIHUANUO	indica	15.4%
S11E0038	BAYUENUO	japonica	40.7%	S11E0187	II-32 B	indica	15.7%
S11E0100	EMATA YIN	japonica	41.0%	S11E0320	TAITUNG 16	indica	15.9%
S11E0288	HUHAN 3	japonica	41.2%	S11E0243	T 2095	indica	16.0%
S11E0059	BICO PRETO	japonica	41.3%	S11E0053	LIUSHA 1	indica	16.0%
S11E0076	MOWANGGU	japonica	42.1%	S11E0035	MAMAGU	indica	16.2%
S11E0246	LIJIANGXINTUANHEIGU	japonica	42.5%	S11E0037	DS001	indica	16.2%
S11E0150	IPEACO 162	japonica	42.8%	S11E0101	CUNGUNUO	japonica	8.9%
S11E0203	ZAOSHUNONGHU 6	japonica	43.0%	S11E0105	BABAILI	japonica	9.3%
S11E0156	MOROBEREKAN	japonica	45.0%	S11E0114	CHIKENUO	japonica	9.3%
S11E0220	HANDAO 8	japonica	45.0%	S11E0131	ZHAXIMA	japonica	11.1%
S11E0133	ITA 141	japonica	45.1%	S11E0132	PUTAOHUANG	japonica	14.0%
S11E0039	HONGKEZHENUO	japonica	49.3%	S11E0147	2428	japonica	14.5%
S11E0225	LAC 23	japonica	54.3%	S11E0193	HAOMAKE(K)	japonica	14.7%
S11E0330	FEIDONGTANGDAO	japonica	67.3%	S11E0307	CYPRESS	japonica	20.2%
means			44.2%				14.1%

Supplementary Table S4 Ratio of the two alleles of the candidate SNPs in deep rooting and shallow rooting varieties from collection 3.

SNP	Deep rooting group	Shallow rooting group	Sig	Chr	Position	Type	Gene name	Gene function
R1	10:0	2:8	0.000**	1	42638216	Intron	LOC_Os01 g73604	LSM domain containing protein
R4	6:4	1:9	0.018*	2	19748822	3’ UTR	LOC_Os02 g33230.1	nucleoside diphosphate sugar epimerase putative expressed
R6	10:0	1:9	0.000**	2	19783893	CDS	LOC_Os02 g33310.1	OsFBD7 D F box and FBD domain containing protein
R7	10:0	2:8	0.000**	2	19813558	CDS	LOC_Os02 g33350.1	conserved hypothetical protein
R11	9:1	9:1	1.000	1	4374717	CDS	LOC_Os01 g08750.2	expressed protein
R12	9:1	8:2	0.556	1	4385983	CDS	LOC_Os01 g08750.2	expressed protein
R14	7:3	1:9	0.004**	1	4391940	CDS	LOC_Os01 g08750.2	expressed protein
R17	10:0	1:9	0.000**	1	4416745	CDS	LOC_Os01 g08800.1	cytochrome P450 putative C expressed
R22	9:1	0:10	0.000**	6	8371625	3’ UTR	LOC_Os06 g14780.2	expressed protein

Based on the results of GWAS mapping, nine candidate SNPs of RDR (ratio of deep rooting) were chosen randomly for further validation by Sanger dideoxy sequencing in 20 extreme RDR varieties. **: means the difference of the skew distribution is very significant (P<0.01); *: means the difference of the skew distribution is significant (P<0.05).

Supplementary Table S5 Comparison of root traits between different subspecies in collection 3.

Subspecies		RDR	DR	H	SR	T	TR	TR/T
Aus	means	33.6%	112.4	96.5	163.5	29.7	343.0	11.9
(3)	minimums	18.9%	60.0	83.5	133.3	24.7	316.5	9.2
	max	43.7%	133.5	107.7	176.5	34.5	362.3	14.7
Indica	means	21.6%	96.6	88.5	261.0	43.8	447.0	10.5
(133)	minimums	8.8%	28.0	69.0	84.8	22.7	149.7	3.5
	max	43.3%	250.5	136.0	503.5	64.3	904.0	23.8
Japonica	means	29.5%	92.3	90.6	166.7	32.1	321.6	10.8
(101)	minimums	8.9%	16.7	65.0	36.3	13.0	65.0	2.4
	max	67.3%	226.3	134.0	477.8	61.0	719.7	20.8
Total	means	25.2%	95.1	89.5	219.2	38.6	391.8	10.6
(237)	SD	8.5%	40.5	13.2	86.2	11.3	131.8	3.6
	minimums	8.8%	16.7	65.0	36.3	13.0	65.0	2.4
	max	67.3%	250.5	136.0	503.5	64.3	904.0	23.8
	Sig[a]	0.000**	0.502	0.273	0.000**	0.000**	0.000**	0.677

The number in parentheses is the amount of varieties used to statistic analysis. a: indicate the significance of variance among the three subspecies, and **: means the difference of the traits among these three subspecies is very significant ($P<0.01$). H: height of the shoot in centimeters; T: number of tillers; DR: number of deep roots; SR: number of shallow roots; TR: total number of roots that penetrate the baskets; RDR: ratio of the deep rooting (=DR/TR); TR/T: number of roots per tiller.

Supplementary Table S6 Correlation coefficients among seven root related traits in all three collections.

	**Correlation**					
	H	T	DR	SR	TR	RDR
T	0.091					
DR	0.176**	0.138**				
SR	0.110	0.508**	0.319**			
TR	0.149**	0.477**	0.587**	0.955**		
RDR	0.071	-0.284**	0.606**	-0.476**	-0.217**	
TR/T	0.269**	-0.428**	0.457**	0.469**	0.546**	0.056

H: height of the shoot; T: number of tillers; DR: number of deep roots; SR: number of shallow roots; TR: total number of roots that penetrate the baskets; RDR: ratio of the deep rooting (=DR/TR); TR/T: number of roots per tiller. **: means the correlation between two traits is very significant (P<0.01).

Supplementary Table S7 Comparison of root traits between upland and lowland rice in collection3.

Means	RDR	H	T	DR	SR	TR	TR/T
Lowland(139)	24.5%	72.8	31.3	53.9	174.3	228.3	7.4
Upland(211)	27.6%	76.4	27.8	56.9	161.9	218.8	8.3
Sig[a]	0.004*	0.000**	0.001**	0.272	0.131	0.319	0.004**

The number in parentheses is the amount of varieties used to statistic analysis. a: indicate the significance of variance among two ecotypes, and **: means the difference of the traits among two ecotypes is very significant ($P<0.01$). H: height of shoot in centimeters; T: number of tillers; DR: number of deep roots; SR: number of shallow roots; TR: total number of roots that penetrate the baskets; RDR: ratio of the deep root (=DR/TR); TR/T: number of roots per tiller.

Supplementary Figure S1 Distribution of RDR (ratio of deep rooting) in RILs. Phenotyping experiments were conducted three times at different locations. a: 2011sh - Shanghai China in 2011; b: 2012hn - Hainan China in 2012 ; and c: 2013hn - Hainan China in 2013.

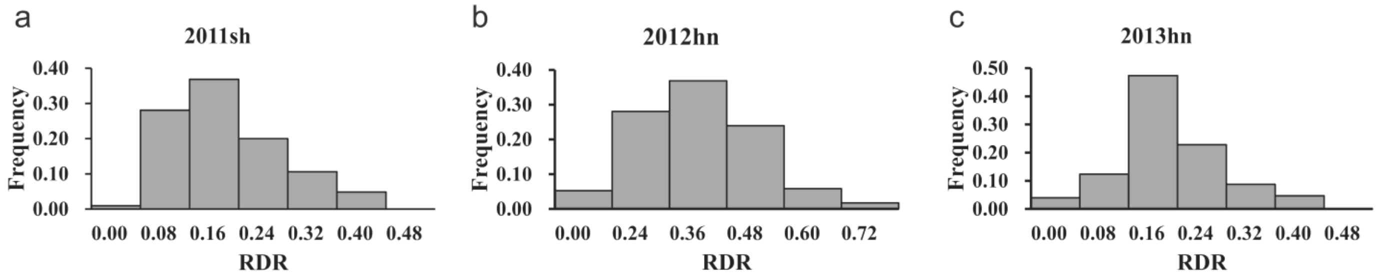

Supplement: Supplementary Data [file supp_erv246_jexbot135657_file001.pdf]
